# Diagnostic Significance of Serum IgG Galactosylation in CA19-9-Negative Pancreatic Carcinoma Patients

**DOI:** 10.3389/fonc.2019.00114

**Published:** 2019-02-27

**Authors:** Ailing Zhong, Ruihuan Qin, Wenjun Qin, Jing Han, Yong Gu, Lei Zhou, Hongqin Zhang, Shifang Ren, Renquan Lu, Lin Guo, Jianxin Gu

**Affiliations:** ^1^Department of Clinical Laboratory, Shanghai Cancer Center, Fudan University, Shanghai, China; ^2^Department of Oncology, Shanghai Medical College, Fudan University, Shanghai, China; ^3^NHC Key Laboratory of Glycoconjugates Research, Department of Biochemistry and Molecular Biology, School of Basic Medical Sciences, Fudan University, Shanghai, China

**Keywords:** pancreatic carcinoma, IgG, galactosylation, diagnostic biomarker, CA19-9

## Abstract

**Background:** Although Carbohydrate antigen 19-9 (CA19-9) is considered clinically useful and informative for pancreatic carcinoma (PC), false positive results, and false negative results have restricted its clinical use. Especially missed or delayed diagnosis of PC patients with negative CA19-9 value limited the utility. To improve prognosis of PC patients, the discovery of reliable biomarkers to assist CA19-9 is desired. Serum IgG galactosylation based on our previous report was altered in PC patients comparing to healthy controls. The objective of this study was to explore the diagnostic significance of IgG galactosylation in assisting CA19-9 for PC in a comprehensive way.

**Methods:** Serum IgG galactosylation profiles were analyzed by MALDI-MS in cohort 1 (*n* = 252) and cohort 2 in which all CA19-9 levels were negative (*n* = 133). In each cohort, not only healthy controls and PC patients but also benign pancreatic disease (BPD) patients were enrolled. Peaks were acquired by the software of MALDI-MS sample acquisition, followed by being processed and analyzed by the software of Progenesis MALDI. IgG Gal-ratio, which was calculated from the relative intensity of peaks G0, G1, and G2 according to the formula (G0/(G1+G2×2)), was employed as an index for indicating the distribution of IgG galactosylation.

**Results:** The Gal-ratio was elevated in PC comparing with that in non-cancer group (healthy controls and BPD). The area under the receiver operating characteristic curve (AUC) of IgG Gal-ratio was higher than that of CA19-9 (0.912 vs. 0.814). The performance was further improved when Gal-ratio and CA19-9 were combined (AUC: 0.928). Meanwhile, Gal-ratio also had great diagnostic value with a sensitivity of 92.31% (AUC: 0.883) in detection of PC at early stage. Notably, IgG Gal-ratio has great sensitivity (90.63%) and specificity (76.81%) in CA19-9-negative PC patients.

**Conclusions:** IgG Gal-ratio had a great performance in detection of PC and could be used to assist CA19-9 in improving diagnosis performance through early stage detection, differentiation from BPD, and PC diagnosis with CA19-9-negative level.

## Introduction

Pancreatic carcinoma (PC) is one of the most lethal tumors, which is predicted to be the second most fatal cancer by 2030 in some countries (1). The 5 year-survival rate for all patients with PC is <5% (2). Survival is better for those with PC diagnosed at its localized stage, because surgical resection offers the only chance of cure at present (3). More than 80% of patients are not detected with PC until the late stage due to missed or delayed diagnosis (4).

In term of diagnosis, carbohydrate antigen 19-9 (CA19-9) is currently the most important serological biomarker (5). As a golden serum biomarker, CA19-9 is widely used for diagnosis, monitoring and prognosis of PC. However, it still suffered from insufficient sensitivity (69–98%) and specificity (46–98%) (6, 7). One reason is that CA19-9 is usually minimally elevated in early premalignant disease, and is elevated in other benign conditions and multiple cancer types (8, 9). Another reason is not all PC patients secrete CA19-9, because ~5–10% of the population with Lewis^a−b−^ has no or scarce secretion of CA19-9 (10). Taken together, it is critical to discover a novel biomarker that not only complement CA19-9 to improve both its sensitivity and specificity before PC progresses to advanced stage, but also is applicable for CA19-9-negative patients.

Glycosylation is one of the most significant post-translational protein modifications. It has been shown to be involved in various pathophysiological steps of tumor development and progression, regulating tumor cell proliferation, invasion, metastasis, angiogenesis as well as immune response (11). Some of serum glycan alternations have been recognized as potential biomarkers in numerous kinds of cancers including PC (12–15). CA19-9 is actually a glycan biomarker, which detects the epitope of sialyl Lewis glycan antigens on mucins and other adhesive molecules (16). Most of recently reported potential serum diagnostic markers of PC are also glycoproteins or glycans, such as CA125, CEA, MUC1, haptoglobin fucosylation (6, 17, 18). However, good results in larger and more comprehensive validations are urgently needed to determine the clinical utility of these reported markers. In our previous study, we found that a decreasing level of galactosylation of serum IgG was associated with PC (19). In this work, we extended the study to investigate and validate its potential as a biomarker in discrimination of PC from benign pancreatic disease (BPD) including pancreatitis and benign pancreatic cysts, early stage PC detection and diagnosis of PC with negative CA19-9 level.

To this aim, we included samples from BPD patients together with healthy controls to compare with those from PC patients. Notably, another set with CA19-9-negative samples whatever they are PC, BPD, or healthy control was specifically enrolled and used to evaluate the diagnostic performance of IgG galactosylation for PC with negative CA19-9 level.

## Materials and Methods

### Study Population and Sample Collection

**Figure 1:**
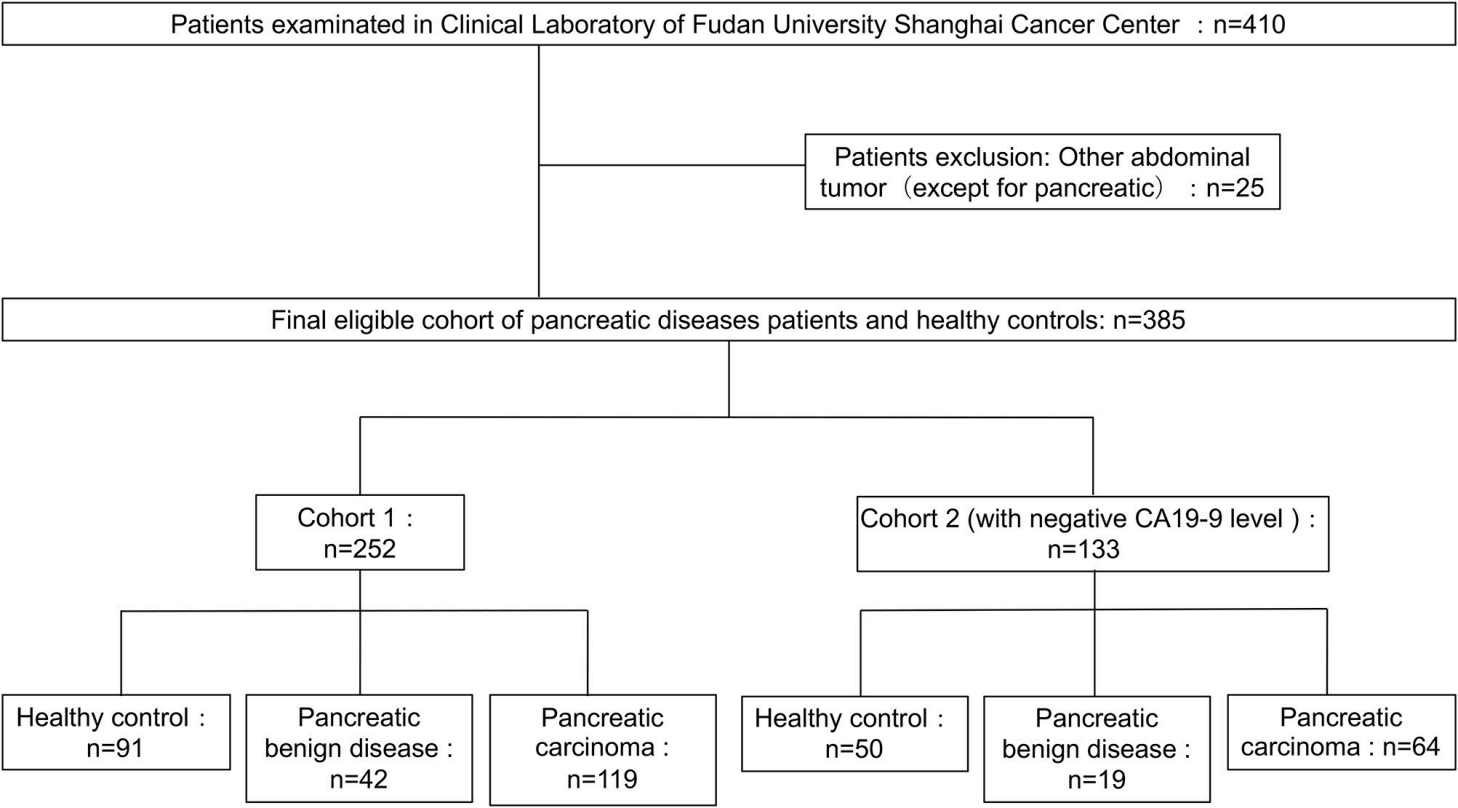
Diagram of study population and cohort selection, with exclusion criteria described on the right-hand side of the diagram.

The 410 serum samples were collected from Shanghai Cancer Center of Fudan University (Shanghai, China). And the serum layer was collected and stored at −80°C until analysis. No more than three cycles of freezing/thaw were allowed for any sample. CA19-9 level was determined on a Roche E170 modular with matched reagents. The exclusion criteria and study cohort flow diagram are summarized in Figure 1. The sera used in cohort 1 and cohort 2 in which all CA19-9 levels were negative (CA19-9 < 37 U/ml) were collected from March 2015 to July 2017. Clinical data from the patients are summarized in Table 1. This study was carried out in accordance with the recommendations of the ethical standards of the Medical Ethics Committee, Fudan University Shanghai Cancer Center. The protocol was approved by the Fudan University Shanghai Cancer Center. All subjects gave written informed consent in accordance with the Declaration of Helsinki.

**Table 1:** Clinicopathological characteristics of all individuals by subgroup.

	**Cohort 1 (*n* = 252)**	**Cohort 2 (*n* = 133)**
**HEALTHY CONTROLS**		
*n*	91	50
Age (years)	59.24	55.06
Male	53 (58.24%)	17 (34.00%)
Female	38 (41.76%)	33 (66%)
CA19-9 > 37 U/ml	12 (13.18%)	0 (0%)
**PANCREATIC BENIGN CYST**		
*n*	27	10
Age (years)	46.19	44.9
Men	4 (14.81%)	2 (20.00%)
Female	23 (85.19%)	8 (80%)
CA19-9 > 37 U/ml	3 (11.11%)	0 (0%)
**PANCREATITIS**		
*n*	15	9
Age (years)	55.8	51.78
Men	12 (80%)	7 (77.78%)
Female	3 (20%)	2 (22.22%)
CA19-9 > 37 U/ml	4 (26.67%)	0 (0%)
**PANCREATIC CARCINOMA**		
*n*	119	64
Age (years)	59.68	57.72
Male	69 (57.98%)	32 (50.00%)
Female	50 (42.02%)	32 (50.00%)
Early stage	26 (21.85%)	18 (28.13%)
Advanced stage	86 (72.27%)	20 (31.25%)
Unknown stage	7 (5.88%)	26 (40.63%)
CA19-9 > 37 U/ml	91 (76.47%)	0 (0%)

### IgG Purification From Human Serum

Purification of IgG was described in previous study (20). IgG from blood serum sample was isolated by the IgG Purification Kit Protein A Spin Plate (Thermo Fisher Scientific, Rockford). The isolation was manipulated according to the manufacturer instructions. Briefly, 100 μl serum was diluted to 200 μl using a proprietary Protein A IgG Binding Buffer. Then the mixture was applied to the protein A plate and washed with 500 μl of Binding Buffer three times to remove unbound proteins. Last, the bound IgGs were eluted with 200 μl of the proprietary IgG Elution Buffer three times in three separate plates. To determine which fractions contained IgGs, the absorbance of each fraction was measured at 280 nm by a bicinchoninic acid (BCA) test (Thermo Fisher Scientific, Rockford.). And the fractions containing IgGs were stored at −20°C until the N-glycan release.

### IgG N-glycans Release and Enrichment

One hundred microliters portion of IgG-containing fractions of Protein A column eluate was used to release N-glycan. According to the previous study, the IgG N-glycans were then released by incubating with PNGase F (New England Biolabs, Inc.) for 12 h at 37°C. The released N-glycans were subsequently purified by porous graphic carbon (PGC) solid-phase extraction. Briefly, a PGC-containing 96-well plate was washed with 200 μl of 0.1% (v/v) trifluoroacetic acid (TFA) in 80% acetonitrile (ACN)/ H_2_O (v/v) and followed by 0.1% (v/v) TFA in H_2_O. The solution of released N-glycans was applied to the PGC-containing 96-well plate three times to allow complete N-glycan adsorption. Then, the plate was washed with H_2_O to remove salts and buffer. The N-glycans derived from IgG were eluted with 100 μl of 0.05% (v/v) TFA in 25% ACN/H_2_O (v/v) and collected for direct MS analysis.

### MALDI MS Analysis

One microliter of collected N-glycans was spotted onto a MALDI target plate (800/384 MTP AnchorChip, Bruker Daltonics, Bremen, Germany) and allowed to dry by air. Then, one microliter 2,5-DHB (10 mg/ml) in 0.1% (v/v) TFA in 50% ACN/H_2_O (v/v) was added onto the sample layer, followed by recrystallized to form homogeneity of the spot surface with ethanol.

Each sample was spotted in triplicate. The samples were interrogated automatically in a “batch mode” by AXIMA Resonance MALDI-QIT-TOF MS (Shimadzu Corp. JP) equipped with a 337 nm nitrogen laser in reflector positive ionization mode. The m/z range was monitored to span from 500 to 5,000. The GlycoWorkbench software was used for the annotation of MS spectra.

### Data Processing and Statistical Analysis

The MALDI-MS spectra data were pre-processed, normalized, and extracted using the software of Progenesis MALDI before further analysis. The following statistical analysis was performed with GraphPad Prism 6 and SPSS Statistics (version 16.0). The normalized volumes from Progenesis MALDI resulting data were aligned and normalized to facilitate identification of possible alterations in the levels of the glycans present. Notably, each serum sample was spotted in triplicate, therefore, three normalized spectra for each serum sample were averaged before statistical tests.

The significance of differences was tested by one-way analysis of variance (ANOVA) *post-hoc* tests with Bonferroni correction using GraphPad Prism 6, and results considered statistically significant when *p*-values were < 0.05. In addition, diagnostic performance of Gal-ratio was further processed by the receiver operator characteristics (ROC) test and generated values of area under the curve (AUC) with 95% confidence intervals (95%CI) using SPSS. If the AUC value was >0.9 that indicates a “highly accurate” test, while values between 0.8 and 0.9 were considered to be “accurate.” When the AUC value was between 0.7 and 0.8, the test was concluded to be “moderately accurate.” An “uninformative” test resulted in an AUC value that was between 0.5 and 0.7.

## Results

### Serum IgG N-glycan Profiles

**Figure 2:**
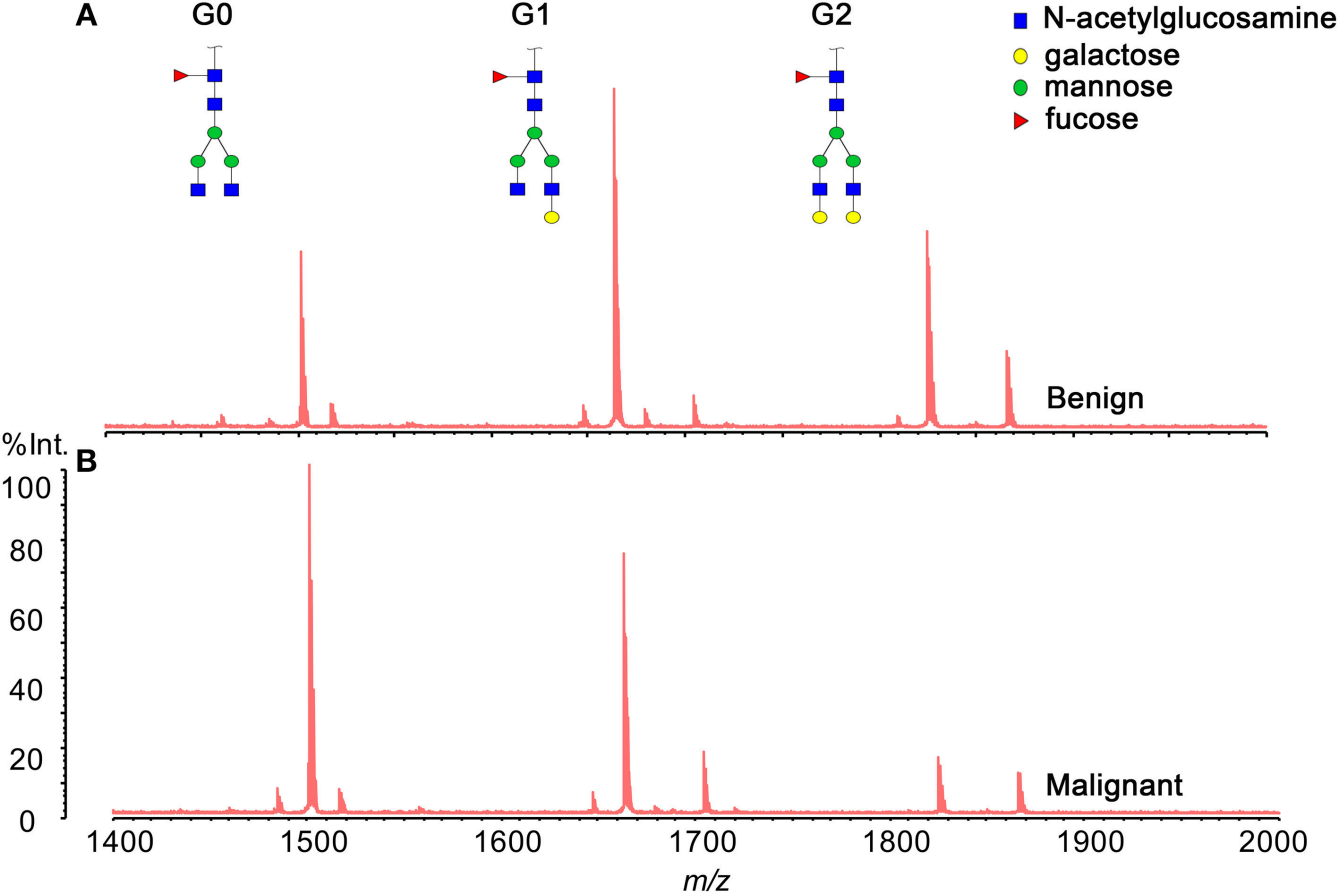
Representative MALDI MS spectra of serum IgG N-glycan profiles acquired for a patient **(A)** with benign pancreatic diseases (BPD) and **(B)** with pancreatic carcinoma (PC).

Three hundred and eighty-five serum samples collected in final eligible cohort were consisted of three groups of participants: healthy controls, patients with BPD, and patients with PC (Figure 1; Table 1). For each group, the age and gender were matched as far as possible. The IgG N-glycans were profiled by MS in both cohort 1 and cohort 2 in which all CA19-9 levels were negative. The typical IgG glycomics profile was shown in Figure 2, which was a representative MALDI mass spectrum for serum IgG N-glycans of both benign and malignant diseases. The level of IgG galactosylation (referred to as Gal-ratio) was calculated from the relative intensity of agalactosylated (G0) vs. monogalactosylated (G1) and digalactosylated (G2) fucosylated biantennary glycans according to the formula of G0/(G1 + G2 × 2) as we previously described (21).

### Performance of Gal-Ratio in Discriminating PC From BPD

We previously showed that the Gal-ratio was significantly higher in PC patients than in healthy controls (19). Besides healthy controls, it is also difficult to discriminate malignant pancreatic cyst lesions from BPD, especially benign pancreatic cyst, due to the only clinical serum biomarker, CA19-9, was not elevated in all of malignant pancreatic cysts (22).

**Figure 3:**
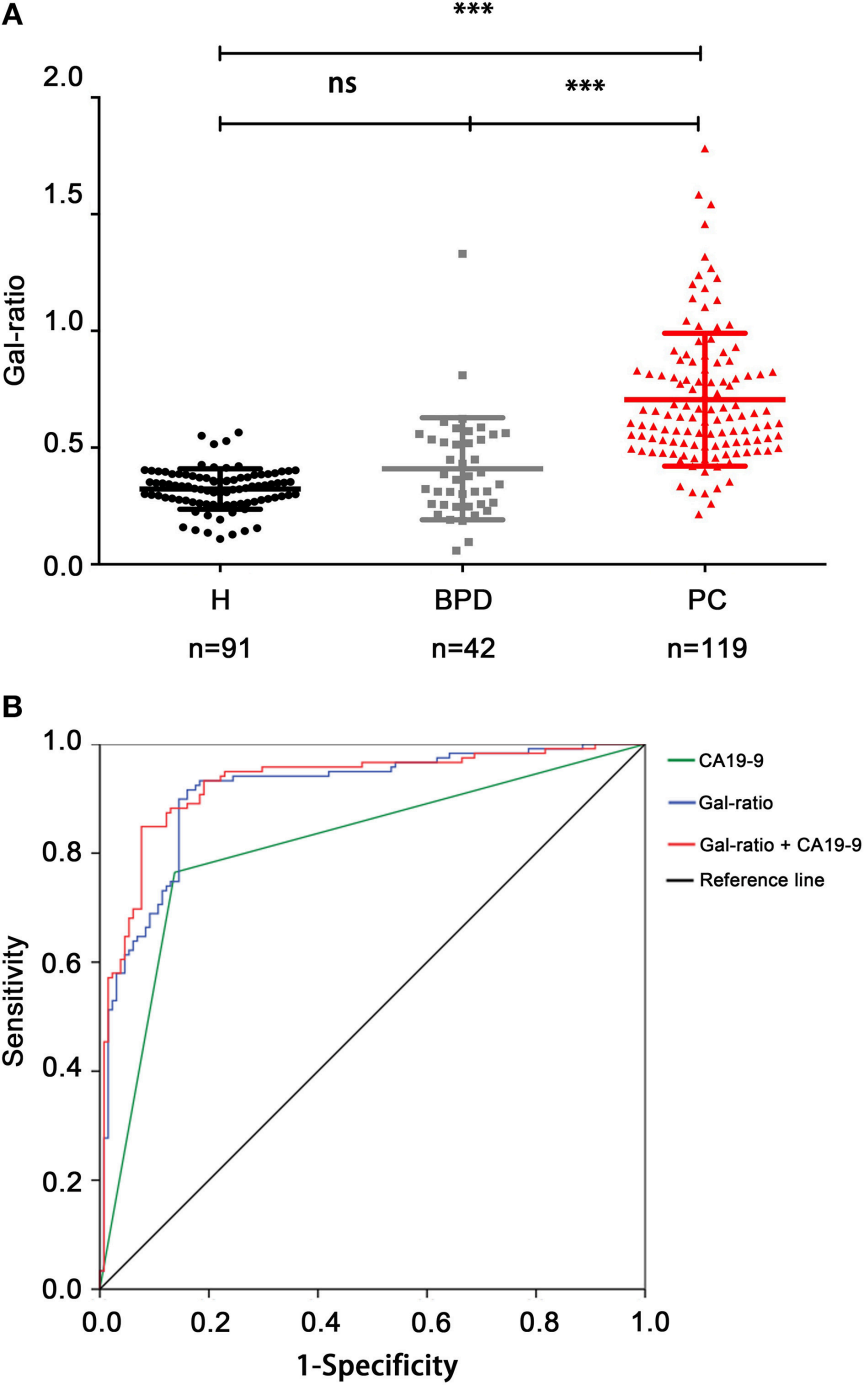
IgG Gal-ratio shows good diagnostic efficacy in identifying PC. **(A)** The comparison of Gal-ratio in healthy controls, benign pancreatic diseases (BPD), and pancreatic carcinoma (PC) (***0.001). **(B)** ROC (Receiver Operating Characteristic) curve for PC diagnosis.

In order to explore whether IgG Gal-ratio can differentiate PC from BPD, IgG Gal-ratio was assayed and compared in 91 healthy controls, 42 patients with BPD, and 119 patients with PC. We found that there was significantly difference between PC and BPD, similar to the result when comparing PC patients to healthy controls (*p* < 0.001, Figure 3A). As far as we know, this is the first time that Gal-ratio has been investigated in BPD samples including benign pancreatic cysts and pancreatitis. This result suggested that IgG Gal-ratio might be used to distinguish benign and malignant pancreatic disease and reduce clinical misdiagnosis.

Since the Gal-ratio of BPD was found similar to healthy controls (Figure 3A), we combined BPD and healthy controls as non-cancer group (*n* = 133) for subsequent study. Next the ROC curve was used to evaluate the performance of IgG Gal-ratio in discriminating malignant tumors from non-cancer group. According to the results, 0.44 was took as the cut off value for Gal-ratio. AUC of Gal-ratio is 0.912 (95%CI: 0.874–0.949) with a high sensitivity of 90.76% and a specificity of 84.21%, which is much greater than that of CA19-9 (AUC: 0.814, 95%CI: 0.757–0.87) with a sensitivity of 76.47% and a specificity of 85.71%. The performance was further improved when Gal-ratio and CA19-9 were combined (AUC: 0.928, 95%CI: 0.894–0.962) (Figure 3B).

### Performance of Gal-Ratio in Detection of PC at Early Stage

**Figure 4:**
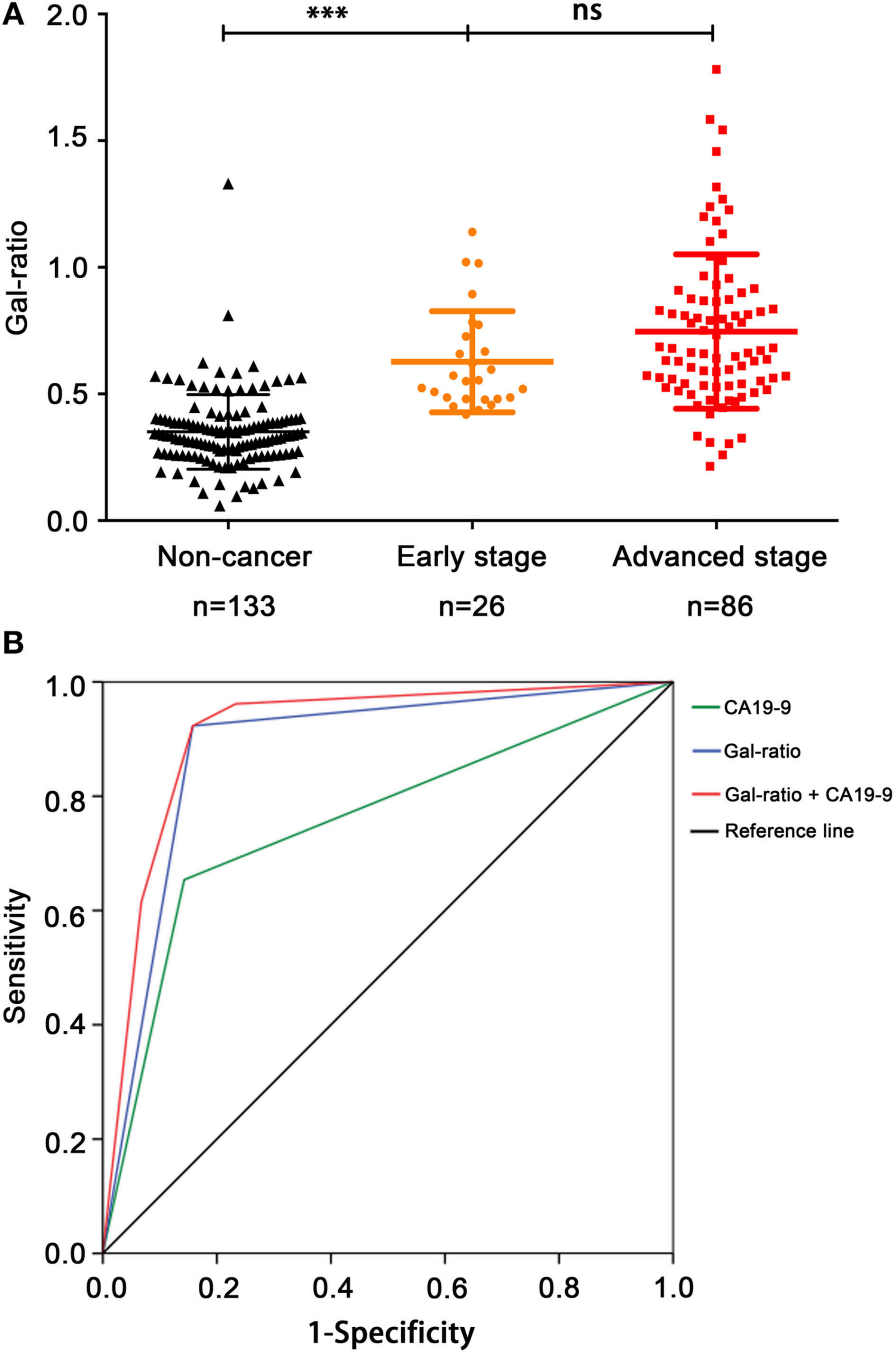
IgG Gal-ratio shows good diagnostic efficacy in identifying PC at early stage. **(A)** The comparison of Gal-ratio in non-cancer control, early stage, and advanced stage of pancreatic carcinoma (***0.001). **(B)** ROC curve for diagnosis of pancreatic carcinoma at early stage.

According to the typical cut off value of 37 U/ml, the positive rate of CA19-9 was 65.38% in early stage PC of our cohort (17/26). Thus, the Gal-ratio difference between non-cancer group and early stage of PC was analyzed to evaluate whether the IgG Gal-ratio has potential to detect early stage PC. The results showed that Gal-ratio was significantly evaluated in early stage of PC, and there was no significant difference between early stage (*n* = 26) and advanced stage PC patients (*n* = 86) (Figure 4A). It is interesting to note this change has already presented in the early stage of PC. Thus, IgG Gal-ratio has potential to be used as a biomarker for diagnosis of early stage PC. We compared the diagnostic value in early stage PC between Gal-ratio and CA19-9 using ROC curves. The AUC of Gal-ratio, CA19-9 and combination of Gal-ratio with CA19-9 were 0.883 (95%CI: 0.813–0.953), 0.755 (95%CI: 0.641–0.870), and 0.913 (95%CI: 0.856–0.971), respectively (Figure 4B). The positive rate of Gal-ratio was 92.31% (24/26), which was much better than that of CA19-9 (65.38%). These results indicated IgG Gal-ratio had similar diagnostic performance for early stage and advanced stage PC. Thus, Gal-ratio may be used for assisting CA19-9 in the diagnosis of early stage PC.

### Performance of Gal-Ratio in Detection of PC in CA19-9-negative Samples

To confirm our findings that Gal-ratio had potential to complement the drawback of high false negative diagnostic results using CA19-9, we further tested Gal-ratio in an independent validation cohort including 50 healthy controls, 19 patients with BPD, and 64 patients with PC when all the samples with negative CA19-9 levels (CA19-9 < 37 U/ml).

In this cohort, we found there was still a significant difference between PC and BPD (*p* < 0.001), similar to the result in cohort 1 (Supplementary Figure 1A). CA19-9 was confirmed no value in the detection of PC in the cohort 2 (sensitivity: 0, Table 2). IgG Gal-ratio had a higher sensitivity and had a potential to be applied in the management of PC with negative CA19-9 level (sensitivity: 90.63%, Table 2). The AUC value of 0.837 presented an accurate performance in distinguishing PC from non-cancer group (BPD and healthy control) (95%CI: 0.765–0.909) (Supplementary Figure 1B). Thus, using normal subjects and patients with BPD as controls, Gal-ratio demonstrated a consistent high diagnostic performance in PC with negative CA19-9 level as in cohort 1.

**Table 2:** The sensitivity and specificity of CA19-9 and Gal-ratio in two cohorts.

	**Cohort 1 (*****n*** **= 252)**		**Cohort 2 (*****n*** **= 133)**	
	**CA 19-9**	**Gal-ratio**	**CA 19-9**	**Gal-ratio**
Sensitivity	76.47% (91/119)	90.76% (108/119)	0 (0/64)	90.63% (58/64)
Specificity	85.71% (114/133)	84.21% (112/133)	100% (69/69)	76.81% (53/69)

## Discussion

CA19–9 is currently the best biomarker for pancreatic carcinoma. However, false negative results (as in Lewis negative or early stage patients) and false positive results (caused by benign pancreatic diseases) limit its clinical application (8), which were confirmed in this study. Therefore, it is urgently needed to explore new biomarkers to make the test more conclusive for the diagnosis of PC patients.

Serological glycomics analysis is an emerging screening tool to find potential biomarkers. To date, glycan alternations in PC have been reported in several studies. Zhao et al. (23) detected 12 N-glycans in subjects of PC, pancreatitis, and healthy controls and the level of total core fucose residues was significantly increased in PC using DSA-FACE technology. Besides total serum glycomics analysis, the glycomics analyses of specific glycoprotein have been reported. Drabik et al. (24) reported four proteins (LIFR, CE350, VP13A, HPT) found in 76 PC patients carrying aberrant glycan structures as compared to those of controls. Nakano et al. (18) found total fucosylated di-, tri-, and tetra-branched glycans of haptoglobin increased in the sera of PC patients compared with choric pancreatitis patients. Remmers et al. (17) indicated aberrant O-glycosylation of mucin was related to the PC progression.

IgG is the most abundant representative of immunoglobulins, with serum concentrations of 10 mg/ml (25). Aberrant IgG glycosylation has been reported to be associated to several cancers including PC (15, 20, 23, 26). In our previous study, we found a significant decrease in IgG terminal galactosylation in many cancers, and put forward a formula “Gal-ratio” which had good performance in PC diagnosis (19). However, the level of terminal galactosylation of total IgG in patients with benign pancreatic cysts or pancreatitis have never been investigated. Notably, benign pancreatic cysts are difficult to be distinguished by imaging examinations. A misdiagnosis would bring unnecessary surgery and psychological burden to benign patients. In this study, to our knowledge, we investigated the IgG terminal galactosylated N-glycans in BPD for the first time and found that Gal-ratio showed no significant difference in BPD and healthy control and demonstrated high potential in discrimination of PC from non-cancer group.

In addition, considering the low true positive rate and high false negative rate, we emphasized on evaluating and validating the diagnostic performance of Gal-ratio in CA19-9-negative cohort in this study. CA19-9-negative PC patients usually include Lewis antigen negative individuals and some patients at early stage. Luo et al. (6) reported that CEA and CA125 had a potential to be applied as biomarkers in Lewis negative patients with PC with sensitivity of 63.8% (CEA) and 51.1% (CA125) compared to 19.1% of CA19-9. Lin et al. revealed that apolipoprotein A-I and transferrin were significant different between CA19-9-negative pancreatic ductal adenocarcinoma and healthy controls through the method of ITRAQ-based quantitative proteomics (27) and suggested that they might be potential biomarkers to be validated in a larger sample cohort. In this work, we provided a potential glycan biomarker, IgG Gal-ratio, to complement those potential protein biomarkers in previous reports. IgG Gal-ratio could detect PC from non-cancer group with the sensitivity of 90.76% in cohort 1. A unique aspect of our study was that our CA19-9-negative validation cohort (cohort 2) where all the samples with negative CA19-9 level were randomly recruited, including PC, healthy, and BPD individuals. Gal-ratio can detect the PC with the positivity rate of 90.63% in this cohort. The finding was of especially great significance in the improvement of discrimination of PC from benign tumor with tumor mass burdens that are usually difficult to accurately diagnose. The results in our study demonstrated that the similar alteration of IgG Gal-ratio at early stage of PC compared to late stage. In addition, the alteration was not affected by the Lewis genotype of populations. These features enabled it as a great potential complementary biomarker to CA19-9. Thus, optimal treatments could be made for patients based on the accurate diagnosis.

IgG glycans seem to have a particularly important role in the immune system and the alternative glycosylation of asparagine 297 in the Fc region of IgG strongly affect its effector functions (28). Agalactosylation can increase the binding with mannose-binding lectin, resulting in promotion of CDC activity, while galatosylation can promote the association between IgG and Fcγ inhibitory receptors, resulting in an increase anti-inflammatory activity (29). Change of IgG galactosylation was first reported in rheumatoid arthritis (RA) (30) and later also reported in other inflammatory diseases, such as inflammatory bowel disease (IBD) (31). In addition, IgG glycans was also reported correlate with type 2 diabetes (32), obesity (33), dyslipidaemia (34), and hypertension (35) besides cancers. These diseases have their own characteristic symptoms and diagnostic methods, enabling the discrimination from cancers. However, it is interesting that similar alterations of IgG galactosylation were observed in these diseases and cancer. And the potential mechanisms and causes of the alteration of IgG Gal-ratio remains unknown. It has been reported that the interaction between cancer cells and immune cells in their microenvironment plays a pivotal role in PC (36). Thus, we hypothesize that the alteration of IgG galactosylation may affect the immune surveillance of PC through determining the interaction of cancer cells and immune cells, which needs further investigated in the future.

In conclusion, we demonstrated that IgG Gal-ratio could assist CA19-9 for PC diagnosis in a more comprehensive way by improving the performance in early stage detection of PC, differentiation malignant tumor from benign diseases as well as detection of PC patients with negative CA19-9 levels. Therefore, IgG Gal-ratio could be applied as a biomarker to assist CA19-9 in reducing false negative results as well as false positive results. Further studies are still needed to validate the potential of Gal-ratio and identify the role of IgG galactosylation in pancreatic carcinoma.

## Author Contributions

RL and SR conceived and initiated this study. SR and RQ designed the experiments and interpreted the results and drew the conclusions. AZ and RL designed the statistical and bioinformatic analyses. AZ, LZ, and HZ collected samples and provided clinical information. RQ, WQ, JH, and YG carried out glycan profiling. AZ and RQ performed the computational analyses. RQ with significant input from SR, AZ, RQ, and WQ wrote the initial draft of the manuscript, which was reviewed by coauthors, including LG and JG.

### Conflict of Interest Statement

The authors declare that the research was conducted in the absence of any commercial or financial relationships that could be construed as a potential conflict of interest.

## Supplementary Material

Supplementary Figure 1 IgG Gal-ratio shows good diagnostic efficacy in identifying PC with negative CA19-9 level. **(A)** The comparison of Gal-ratio in healthy controls, benign pancreatic diseases (BPD) and pancreatic carcinoma (PC) (^***^0.001). **(B)** ROC curve of Gal-ratio for PC diagnosis.
