# Supplementary figures and images for: Diagnostic Significance of Serum IgG Galactosylation in CA19-9-Negative Pancreatic Carcinoma Patients
Source: Front Oncol. 2019 Feb 27;9:114. doi: 10.3389/fonc.2019.00114 (PMC6402387; doi:10.3389/fonc.2019.00114)

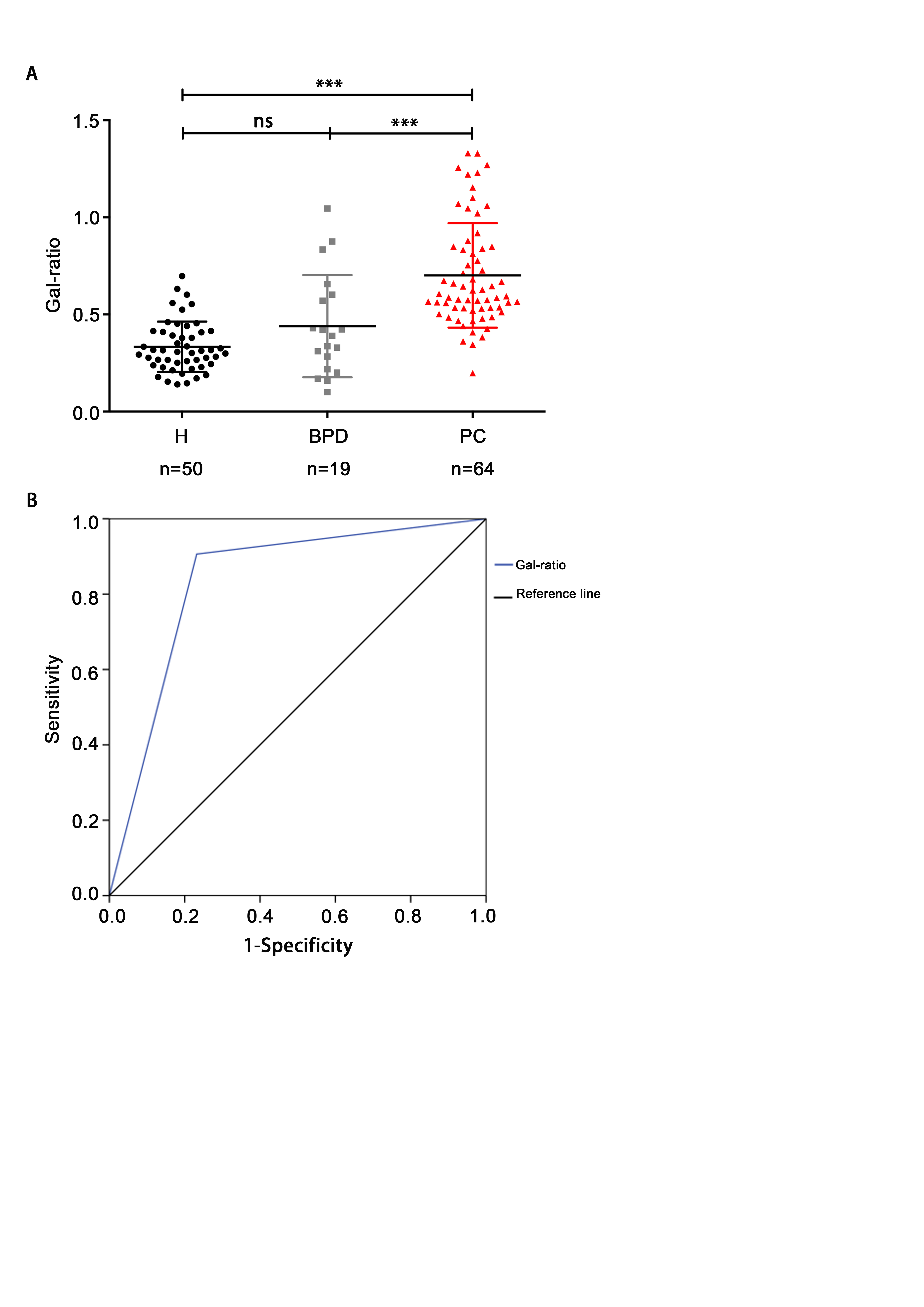

Supplement: Supplementary Figure 1 — IgG Gal-ratio shows good diagnostic efficacy in identifying PC with negative CA19-9 level. (A) The comparison of Gal-ratio in healthy controls, benign pancreatic diseases (BPD) and pancreatic carcinoma (PC) (***0.001). (B) ROC curve of Gal-ratio for PC diagnosis. [file Image_1.TIF]
